# Establishing abdominal height cut-offs and their association with conventional indices of obesity among Arab children and adolescents

**DOI:** 10.4103/0256-4947.62835

**Published:** 2010

**Authors:** Nasser Al-Daghri, Majed Alokail, Omar Al-Attas, Shaun Sabico, Sudhesh Kumar

**Affiliations:** a From the College of Science, King Saud University, Riyadh, Saudi Arabia; b From the Warwick Medical School, Warwick, United Kingdom

## Abstract

**BACKGROUND AND OBJECTIVES:**

Obesity, particularly childhood obesity is common in the Middle East, but no studies have examined the relationship of sagittal abdominal diameter (SAD) or abdominal height to conventional markers of obesity in this region. This is the first study to document the association of SAD with measures of obesity among Arab children and adolescents.

**METHODS:**

Nine hundred sixty-four Saudi children aged 5-17 years (365 prepubertal, including 146 boys and 219 girls; 249 pubertal, including 125 boys and 124 girls; and 350 postpubertal, including 198 boys and 152 girls) were included in this cross-sectional study.

**RESULTS:**

SAD was significantly correlated with indices of obesity regardless of gender, but was strongest among pubertal boys. The cut-off values were as follows: for prepubertal children, 14 cm (equivalent to 50th percentile among girls and 60th percentile among boys); for pubertal children, 15 cm for girls (30th percentile) and 16 cm for boys (50th percentile), and for postpubertal, 21.5 cm for girls (70th percentile) and 22 cm for boys (80th percentile).

**CONCLUSION:**

SAD is a reliable indicator of visceral obesity among Arab children and adolescents in particular. Prospective studies should be done to determine whether such an association translates to a promising risk factor for hard endpoints such as diabetes mellitus and coronary heart disease.

By definition, obesity is a disproportionate buildup of energy in the form of body fat, which impairs health. The degree of health impairment is determined by the amount and distribution of fat, and the presence of other risk factors. Among the mentioned factors, it is the distribution of fat that serves as key to understanding the relationship between obesity and disease.^1^ The significance of body fat distribution in relation to various metabolic diseases is not a new concept. Studies carried out by the late Dr. Jean Vague showed that insulin resistance and hyperinsulinism are the metabolic bases for accelerated atherosclerosis, diabetes, gout, and uric calculi in the android type of obesity.^2^ These insights have imposed a need to determine body composition, and the mass and fat tissue distribution.

Body mass index (BMI) and waist-hip ratio (WHR) are two of the most commonly used indices for obesity. In terms of their relation to adverse coronary outcomes, however, WHR is more consistent as an index of abdominal obesity and a stronger predictor of atherosclerotic events compared to BMI^3^ in both men^4^ and women.^5^ A particularly important anthropometric parameter which has been increasingly applied in the recent years is the sagittal abdominal diameter (SAD) (also called abdominal height) measured by calipers constructed by Kahn.^6^ It has recently gained attention because of its stronger correlation to cardiovascular diseases compared to the other anthropometric measures.^7^ In a recent study by Sampaio et al, SAD was considered to be a valid predictor of visceral abdominal fat with very high reliability (interclass coefficient, 0.99).^8^ Although this anthropometric indicator measures only the visceral fat tissue, it gives a very good estimate of metabolic risks because of the type of fat tissue involved.

Few studies have actually linked SAD to other metabolic abnormalities. A recent study by Mukuddem-Petersen and colleagues concluded that the use of SAD had no advantages over simpler and more commonly used anthropometric measures such as the waist circumference in older men and women.^9^ On the other hand, Petersson and colleagues emphasized that SAD identifies insulin resistance, subclinical inflammation, and hyperlipidemia, at least among Swedish women and women of Middle eastern descent.^10^ No such study has been undertaken in the Middle East, particularly in Saudi Arabia, where the incidence of obesity, including childhood obesity, and other metabolic risk factors are high. Furthermore, very few studies have been undertaken with regards to SAD among children. Hence, this study aims to establish cut-off points for SAD (values below the cut-off are considered abnormal) among Arab youth and to determine the relationship of SAD to conventional markers of obesity such as BMI, waist, hip circumference, and WHR.

## METHODS

This cross-sectional study was conducted at the Diabetes and Endocrinology Research Laboratory of King Saud University, Riyadh Kingdom of Saudi Arabia from January 2008 to December 2008. The subjects were Saudi children aged 5-17 years who were recruited randomly by primary care physicians through door-to-door interviews within Riyadh, Saudi Arabia. Recruitment was part of the ongoing biomarker screening of the Ministry of Health in collaboration with King Saud University. Written and informed assent from the children and parental consent were obtained. Ethical approval was obtained from the Ethics Committee of the College of Medicine and Research Center, King Saud University Riyadh, Kingdom of Saudi Arabia.

The 964 subjects included 365 prepubertal (146 boys and 219 girls), 249 pubertal (125 boys and 124 girls), and 350 postpubertal (198 boys and 152 girls) children. Tanner staging was used to assess the pubertal stage of boys and girls by trained physicians during their physical exam. Subjects within stage 1 were assigned to the pre-pubertal stage, subjects within stages 2-4 to the pubertal stage, and subjects in stage 5 to the post-pubertal stage. Children with acute or chronic medical conditions as well as those with diabetes and/or asthma were not included in the study. A generalized datasheet containing demographic information and consent was given to the subjects with their parents' assistance along with a sheet for anthropometric measurements.

Trained research nurses were assigned to record anthropometric indices. A measurement called percentile of BMI was used to identify overweight and obesity in children and adolescents.^11^ The BMI used for this study was gender- and age-specific for children. Measurements included height, weight, waist circumference, hip circumference, and SAD. Body weight in light clothes was measured to the nearest 0.1 kg using a standardized Detecto balance beam scale (Detecto Scale Inc, Brooklyn, NY, USA). Height was assessed to the nearest 0.5 cm using standardized stadiometers (Detecto Scale Inc, Brooklyn, NY, USA). Subjects were asked to stand upright on a flat surface without shoes, with the back of their heels and the occiput on the stadiometer. BMI was calculated as weight (kg) divided by height in squared meters. Waist circumference was measured using a standardized measuring tape to the nearest centimeter, taken midway between the lowest rib and iliac crest, whereas hip circumference was measured at the level of the greater trochanters. Holtain Khan abdominal calipers (Holtain Ltd, Crymych, UK) were used to measure SAD. Each subject was examined in the supine position on a firm examination table. Using sliding calipers with parallel blades, a direct reading could be made between the lower arm (touching the subject's back) and the sliding upper arm (touching the front of the subject's abdomen). The measurement was taken after normal expiration (as instructed by the research nurse) with the subject in a relaxed position, and the caliper not indenting the subject's skin. Systolic and diastolic blood pressure readings were also measured using an aneroid sphygmomanometer (Omron with pediatric cuffs by Kappa Medial LLC, AZ, USA).

The data were analyzed using the Statistical Package for the Social Sciences (SPSS for Windows version 11.5). Data are expressed as mean (standard deviation) or as median and range if not normally distributed. Group comparisons were done using the t test or by the Mann-Whitney U-test if not normally distributed. Frequencies were expressed in percentages. Normalization of BMI was done to calculate *z* scores. BMI *z* scores were plotted according to the SAD percentiles of boys and girls that had been stratified to pubertal stage. In essence, the *z* score represents the number of SD units by which a given score deviates above or below the mean score. As zero corresponds to the mean BMI, the percentile SAD value nearest to a *z* score of zero was deemed to be the cut-off value. Simple and partial correlation coefficients were determined and regression analysis done to determine relationships between variables of interest. Significance was set at *P*<.05.

## RESULTS

**Figure 1:**
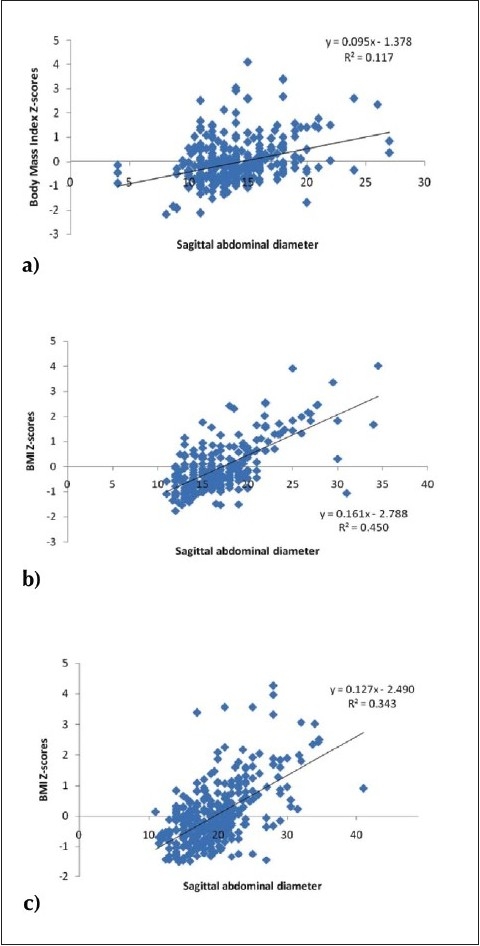
Association of SAD with BMI z scores according to pubertal stage: a) prepubertal, b) pubertal and c) postpubertal.

**Figure 2:**
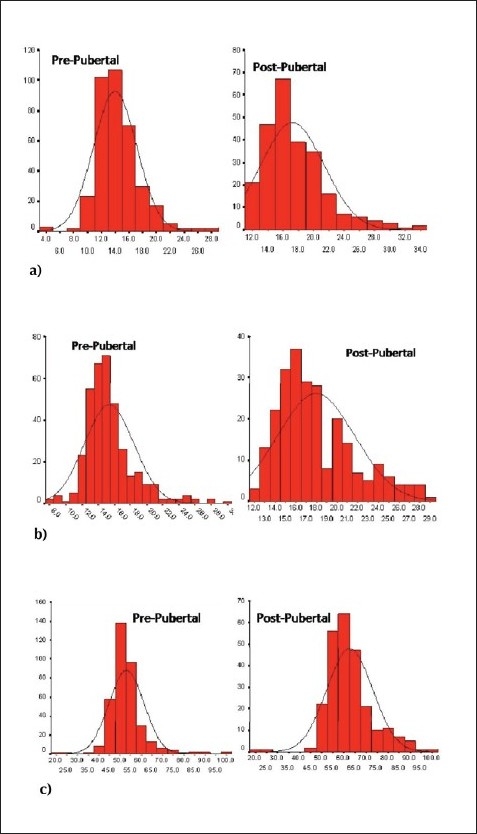
Histograms of selected anthropometric measurements according to pubertal stage: a) SDI Pprepubertal vs postpubertal, b) BMI prepubertal vs postpubertal and c) waist circumference distribution vs postpubertal.

**Figure 3:**
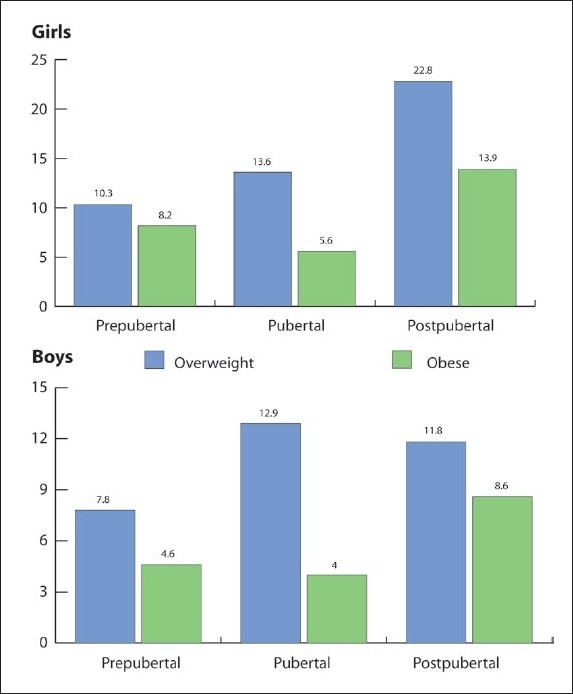
Prevalence of obesity according to pubertal stage.

As expected, all parameters of postpubertal subjects were significantly higher compared to their pubertal and prepubertal counterparts, regardless of gender (Table 1). The cut-off values for SAD are shown in red in Table 2, which shows BMI *z* scores relative to SAD percentiles (Table 2). Although there was a significant correlation between SAD and the indices of obesity, regardless of gender, also noteworthy was the very strong association of SAD with BMI as well as waist and hip circumference of pubertal boys (R values: 0.77, 0.75, 0.74; *P*<.001 respectively). Furthermore, there was a weak association between systolic blood pressure and SAD (R = 0.18; *P*<.05) among pubertal boys that was not present in other subgroups (Table 3). This association was lost after adjusting for BMI, waist, and hip circumference. The strongest association of SAD was with BMI, regardless of gender, during the pubertal stage (R2 = 0.45; *P*<.001) (Figure 1). Neither BMI nor SAD values of postpubertal subjects showed a Gaussian distribution (Figure 2), which probably explains why the cut-off points established for both boys and girls in this age group were beyond the 50th percentile. The prevalence of overweight and obesity in both boys and girls which was most evident in the postpubertal group (Figure 3).

**Table 1:** Anthropometric characteristics of subjects according to pubertal stage.

Parameter	Prepubertal (n=365)	Pubertal (n=249)	Postpubertal (n=350)
Age (years)			
Girls	7.6 (1.8)^a^^b^	12.0 (0.8)^b^	15.5 (1.1)
Boys	7.5 (1.8)^a^^b^	12.2 (0.8)^b^	15.4 (1.1)
Body mass index (kg/m^2^)			
Girls	15.7 (3.5)^a^^b^	18.5 (3.8)^b^	22.2 (5.1)
Boys	15.4 (3.6)^a^^b^	17.8 (4.5)^b^	20.6 (5.6)
Systolic blood pressure (mm Hg)			
Girls	99.3 (12.3)^a^^b^	104.3 (10.6)^b^	108.6 (10.4)
Boys	99.4 (8.9)^a^^b^	99.5 (13.6)^b^	107.8 (9.8)
Diastolic blood pressure (mm Hg)			
Girls	66.1 (7.2)^a^^b^	68.4 (8.1)^b^	71.1 (8.0)
Boys	65.3 (6.3)^a^^b^	66.2 (10.5)^b^	70.7 (9.1)
Waist (cm)			
Girls	53.1 (9.8)^a^^b^	61.8 (10.4)^b^	68.8 (11.9)
Boys	53.3 (9.9)^a^^b^	63.5 (10.3)^b^	70.3 (12.4)
Hips (cm)			
Girls	63.1 (9.9)^a^^b^	76.3 (13.4)^b^	87.1 (13.6)
Boys	61.5 (7.9)^a^^b^	75.1 (10.6)^b^	85.1 (14.9)
SAD (cm)			
Girls	14.2 (3.4)^a^^b^	17.6 (4.3)^b^	19.5 (4.5)
Boys	13.8 (2.8)^a^^b^	16.9 (3.90)^b^	19.6 (4.7)

Data are mean (standard deviation)

a denotes significance compared to pubertal

b denotes significance compared to postpubertal; significant at *P*<.05.

**Table 2:** Percentiles of sagittal abdominal diameter (SAD) and corresponding body mass index (BMI) z scores of subjects according to pubertal stage.

SAD (cm) percentile	BMI z scores					
	Prepubertal		Pubertal		Postpubertal	
	Girls	Boys	Girls	Boys	Girls	Boys
10	11 (−0.15)	11 (0.03)	13 (−0.1)	12.7 (−1.07)	14 (−0.46)	14.1 (−0.39)
20	12 (−0.08)	11 (0.03)	14 (−0.24)	14 (−0.45)	15.9 (−0.63)	16 (−0.46)
30	12 (−0.08)	12 (−0.09)	15 (0.03)	15 (−0.25)	17 (−0.04)	17 (−0.33)
40	13 (−0.45)	13 (−0.05)	15.7 (−0.71)	15.2 (−0.56)	18 (−0.13)	18 (0.66)
50	14 (−0.01)	13.6 (−0.49)	17 (−0.3)	16 (−0.04)	19 (−0.4)	19 (0.57)
60	14.7 (−0.68)	14 (−0.02)	18 (0.3)	17 (−0.26)	20 (0.33)	20 (0.51)
70	15.4 (−0.19)	15 (−0.17)	19 (−0.36)	18 (−0.25)	21.5 (0.02)	21 (0.48)
80	16.5 (0.14)	16 (−0.05)	20.6 (−0.05)	19.5 (0.63)	23 (0.26)	22 (0.14)
90	18.3 (1.38)	18 (−0.2)	24 (1.44)	22 (2.08)	25 91.3)	28 (3.9)

Data are SAD (z score). Cut-off values for SAD in red.

**Table 3:** Pearson correlation coef.cients using SAD as a dependent variable among subjects in different pubertal stages.

Parameter	Prepubertal		Pubertal		Postpubertal	
	Girls	Boys	Girls	Boys	Girls	Boys
Age (years)	0.18^a^	0.27^b^	0.06	-0.05	0.12	0.23^b^
BMI (kg/m^2^)	0.24^b^	0.22^b^	0.32^b^	0.77^b^	0.50^b^	0.44^b^
Waist (cm)	0.23^b^	0.33^b^	0.33^b^	0.75^b^	0.39^b^	0.41^b^
Hips (cm)	0.30^b^	0.44^b^	0.31^b^	0.74^b^	0.34^b^	0.12
Systolic blood pressure (mm Hg)	0.11	0.01	0.03	0.18^a^	0.06	-0.02
Diastolic blood pressure (mm Hg)	0.12	0.02	-0.01	0.13	0.05	-0.03

a *P*<.05;

b *P*<.001

## DISCUSSION

Anthropometric measures are clinically useful because their collection is noninvasive and cheap. Measures such as BMI are highly associated with cardiovascular disease. On the other hand, measures of abdominal obesity such as the waist-hip ratio could be more clinically useful than BMI to predict diabetes and cardiovascular disease.^10^ SAD has recently gained attention because of its stronger correlation with cardiovascular diseases compared to other anthropometric measures.^11^ In a study done by Anjana and colleagues, both waist circumference and SAD were found to correlate well with visceral and abdominal obesity, both of which are strongly correlated to the development of diabetes.^12^ In another recent study by Oppert et al, SAD was found to be the only significant predictor of cardiac death compared to other measures of obesity.^13^ Kvist et al were the first to demonstrate that the sagittal diameter measured by CT scan was closely related to the volume of visceral fat.^14^ The correlation of the sagittal diameter was 0.94 in 19 women and 0.92 in 24 men, with subjects presenting a wide range of BMI. The correlations between the waist circumference and visceral fat were found to be 0.85 and 0.88, respectively. These correlations are considerably higher than those observed between anthropometric variables and the visceral fat area measured at the level of the umbilicus in obese men and women.^15^

To the best of our knowledge, no study has been conducted to determine SAD cut-offs and their relationship to other measures of obesity among Arab youth. This is particularly important as childhood obesity that persists into adulthood is an inevitable factor for the growing incidence of noncommunicable chronic diseases. Furthermore, there is still no existing consensus as to the definition of pediatric metabolic syndrome.^16^ Compared to other studies, our postpubertal cut-offs were relatively higher compared to those found in Brazilians (19.3 cm and 20.5 cm as the threshold values for SAD in women and men),^8^ suggesting probable ethnic variation or other factors such as choice of the cohort.

In our study, SAD was strongly associated with several components of the metabolic syndrome (BMI and systolic blood pressure) although this does not apply to all ages. This age-wise difference reflects the body's evolution through childhood and puberty and the associated changes in metabolic and clinical characteristics.^16^ Nevertheless, SAD remains a promising alternative measure of central obesity among children as it is superior to other anthropometric measures in terms of visceral fat estimation.^17^ This is because when a person with enlarged intraabdominal adipose tissue is standing, all fat tissue is pulled downwards. Hence, waist or hip circumference measurements may not be as accurate in assessing the intraabdominal fat, especially among children who are very obese. When the same person lies supine, the mass shifts cranially, causing anterior projection of the abdomen which is measured by the sagittal diameter. Thus, it is the anteroposterior fat that seems to be important for the prediction of the metabolic syndrome.^18^

Our results showed gender differences in terms of the strength of SAD's association with other measures of obesity, which was more evident in the pubertal group as compared to the prepubertal group. This confirms the findings of Arfai and colleagues at least in terms of visceral fat.^19^ Furthermore, in our results, boys and girls showed no difference in SAD values, indicating a monomorphic trend in values for intraabdominal adipose tissue at least during childhood. In contrast, visceral fat is more predominant in males due to considerable gender differences in fat topography among adults.^18^

Epidemiological studies conducted in Saudi Arabia point to an increased incidence of overweight and obese Saudi children.^20^–^24^ Given that obesity is an influential risk factor for certain cancers and chronic diseases such as atherosclerosis, hypertension, and most especially, diabetes mellitus (prevalence of 23.7% in 2004 alone^25^), it is imperative to address childhood obesity as critically as one would address its co-morbidities. The use of SAD therefore offers a cheap but reliable index of how close a child is to developing insulin-resistance-related diseases. Establishing SAD cut-offs that are specific to gender and age, is imperative to develop meaningful and evidence-based strategies aimed at reducing risk factors through parental education, school programs, and childhood training.

Although the sample size was large enough to elicit the needed associations, a bigger sample size with the inclusion of rural children would have distinguished the impact of SAD on various age groups as well as establishing more reliable cut-offs that would have been age- and gender-specific. Furthermore, as this study was confined only to anthropometric measurements, further studies which will include other components of the metabolic syndrome such as lipid profile and glucose levels besides other laboratory measurements, are necessary to strengthen the clinical relevance of SAD among the obese pediatric population. A prospective approach is also suggested to determine whether variations in SAD translate to changes in the metabolic profiles of children.

In conclusion, SAD can be used as a reliable indicator of visceral obesity among children and adolescents in particular. Further studies should be done to prospectively compare its association to harder measures such as cardiometabolic risk factors, components of metabolic syndrome, and indices of insulin resistance to determine whether SAD can be a promising risk factor for diabetes mellitus and coronary heart disease.
